# Increasing muscle co-contraction speeds up internal model acquisition during dynamic motor learning

**DOI:** 10.1038/s41598-018-34737-5

**Published:** 2018-11-05

**Authors:** James B. Heald, David W. Franklin, Daniel M. Wolpert

**Affiliations:** 1 Computational and Biological Learning Lab, Department of Engineering, University of Cambridge, Cambridge, CB2 1PZ United Kingdom; 2 Neuromuscular Diagnostics, Department of Sport and Health Sciences, Technical University of Munich, 80992 Munich, Germany; 3 Zuckerman Mind Brain Behavior Institute, Department of Neuroscience, Columbia University, New York, United States

## Abstract

During reaching movements in the presence of novel dynamics, participants initially co-contract their muscles to reduce kinematic errors and improve task performance. As learning proceeds, muscle co-contraction decreases as an accurate internal model develops. The initial co-contraction could affect the learning of the internal model in several ways. By ensuring the limb remains close to the target state, co-contraction could speed up learning. Conversely, by reducing kinematic errors, a key training signal, it could slow down learning. Alternatively, given that the effects of muscle co-contraction on kinematic errors are predictable and could be discounted when assessing the internal model error, it could have no effect on learning. Using a sequence of force pulses, we pretrained two groups to either co-contract (stiff group) or relax (relaxed group) their arm muscles in the presence of dynamic perturbations. A third group (control group) was not pretrained. All groups performed reaching movements in a velocity-dependent curl field. We measured adaptation using channel trials and found greater adaptation in the stiff group during early learning. We also found a positive correlation between muscle co-contraction, as measured by surface electromyography, and adaptation. These results show that muscle co-contraction accelerates the rate of dynamic motor learning.

## Introduction

The reduction of kinematic errors during reaching movements in the presence of novel dynamics occurs through at least two complementary mechanisms^1,2^. First, participants can learn an internal model of the dynamics and use this model to anticipate and compensate for perturbations^3–6^. Second, participants can use a less-specific strategy of stiffening up the limb through muscle co-contraction (i.e., impedance control)^1,7–9^ to reduce the kinematic errors that result from perturbing dynamics^10–12^. Several studies have shown that both these mechanisms contribute to the early compensation for novel dynamics^13–17^ and that muscle co-contraction decreases as the internal model is learned^1,18–20^. Therefore, muscle co-contraction offers a temporary, non-specific strategy to compensate for novel dynamics until they are learned by the internal model. Here we ask whether the increase in muscle co-contraction during exposure to novel dynamics affects the learning of the internal model.

Muscle co-contraction could affect dynamic motor learning in three contrasting ways. First, it could facilitate learning by keeping the state of the limb close to the desired state required for task success (i.e., the target). By resisting force-field displacements, muscle co-contraction increases the alignment between the actual motions experienced and the motion to be learned. Importantly, because participants learn the mapping from visited states, rather than planned states, to dynamics^21^, increased muscle co-contraction should accelerate learning by concentrating adaptation within the region of state space associated with the planned motion. Indeed, in artificial neural networks, it has been shown that the inverse dynamics of a plant can be learned faster when the actual trajectories executed are closer to the desired trajectories to be learned, as this ensures that local error information is applicable to the region of state space to be learned^22^. Conversely, reduced muscle co-contraction should slow down learning by increasing the range of actual motions experienced and thus spreading adaptation more thinly across state space. Consistent with this idea, adaptation to a visuomotor rotation is less complete when the rotation is introduced abruptly compared with gradually^23^.

Second, muscle co-contraction could impede learning by reducing kinematic variability^10,24–26^, which has been shown in some forms to enhance error-based learning, both in humans^27^ and songbirds^28–30^. Moreover, muscle co-contraction reduces kinematic errors, which are a key training signal for internal model acquisition^31–34^. Although some saturation is seen for large errors, in general, trial-to-trial learning increases with the magnitude of the error on the previous trial^35,36^. Therefore, by reducing kinematic errors, muscle co-contraction could reduce trial-to-trial learning and thus decrease the overall rate or extent of learning.

Third, muscle co-contraction could have no effect on learning if kinematic errors are normalized against the current level of muscle co-contraction. Indeed, incoming sensory information is often pre-processed on the basis of motor output. For example, reafferent feedback is attenuated by sensory predictions based on efference copies of motor commands^37–39^. It is therefore possible that error-based learning is driven by pre-processed kinematic errors. Consequently, even though kinematic errors are smaller during muscle co-contraction, the motor system may still adapt to the true internal model performance error. Alternatively, or concurrently, the opposing effects of concentrated learning in state space (hypothesis one) and smaller kinematic errors (hypothesis two) could cancel out such that muscle co-contraction has no effect on learning.

To differentiate between these hypotheses, we developed a novel paradigm in which participants were either pretrained to increase (stiff group) or decrease (relaxed group) muscle co-contraction in the presence of dynamic perturbations. A third group (control group) was not pretrained at all. We then examined force-field adaptation using channel trials, which constrain movements to a straight line and allow us to measure the force generated into the channel walls as a measure of predictive compensation. In the initial stage of exposure, the stiff group adapted more than both the relaxed group and the control group. In both the initial and final stage of exposure, we found a positive correlation between an individual’s level of muscle co-contraction and their level of adaptation.

## Materials and Methods

Thirty-six neurologically intact participants (16 men and 20 women; age 25.3 ± 5.3 yr, mean ± s.d.) were recruited to participate in the experiment, which had been approved by the Cambridge Psychology Research Ethics Committee and was performed in accordance with guidelines and regulations. All participants provided written informed consent and were right-handed according to the Edinburgh handedness inventory^40^. Participants had not previously performed any experiments involving velocity-dependent curl fields.

### Apparatus

**Figure 1:**
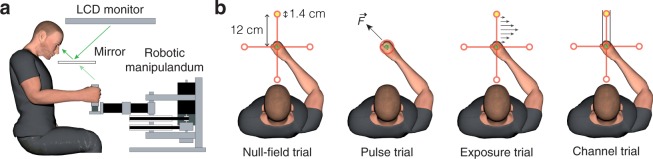
Paradigm. (**a**) The participant grasped the handle of the robotic manipulandum (vBOT) while seated. Visual feedback was presented veridically using a horizontally-mounted monitor viewed through a mirror. (**b**) At the start of each reaching trial, a target was illuminated (yellow circle), cueing the participant to move their hand (green circle) from the center of the workspace to the illuminated target by making a fast reaching movement. In the pulse phase, a series of brief pulses of force (black arrow) was applied to the handle of the manipulandum in a random direction. Participants in the stiff group were instructed to keep their hand within a 1.5 cm radius of the home position (red ring) by co-contracting the muscles of their arm. Participants in the relaxed group were instructed to let their hand move freely by relaxing the muscles of their arm. In the exposure phase, a velocity-dependent force field was applied. Channel trials were used to assess adaptation for the 90° target only.

Experiments were performed using a vBOT planar robotic manipulandum^41^ with virtual-reality system and air table (Fig. 1a). The vBOT is a modular, general-purpose, two-dimensional planar manipulandum optimized for dynamic learning paradigms. Position was measured using optical encoders sampled at 1 kHz. Torque motors allowed forces to be generated at the endpoint. A monitor mounted above the vBOT projected visual feedback into the plane of movement via a horizontal mirror. The location of the mirror prevented direct vision of the hand and forearm. Participants were seated in front of the robotic manipulandum with their torso restrained by a four-point harness to reduce movement. The handle of the manipulandum was grasped with the right hand, while the right forearm was supported on an air sled, which constrained arm movements to the horizontal plane.

Movements were performed in either a null field (vBOT passive), a velocity-dependent curl force field or in a simulated channel. In the curl field, the force generated at the handle of the manipulandum was given by:

1 $$[\begin{array}{c}{f}_{\text{x}}\\ {f}_{\text{y}}\end{array}]=b[\begin{array}{cc}0 & -1\\ 1 & 0\end{array}]\,[\begin{array}{c}\dot{x}\\ \dot{y}\end{array}]$$

where *f*_x_, *f*_y_, $\dot{x}$ and $\dot{y}$ are the forces and velocities at the handle in the *x*- and *y*-directions respectively. The gain *b* was set to ±16 Ns/m, where the sign of *b* specified the direction of the curl (clockwise or counterclockwise). On channel trials, the hand was constrained to move along a straight line from the home position to the target, using stiffness (6,000 N/m) and damping (5 Ns/m) perpendicular to the movement direction. Channel trials clamped the kinematic error to zero, allowing forces that were generated in a feedforward manner to be measured orthogonal to the direction of reach^13,42^.

### Electromyography

Surface electromyography (EMG) was recorded from two monoarticular shoulder muscles (pectoralis major and posterior deltoid), two biarticular shoulder-elbow muscles (biceps brachii and the long head of the triceps) and two monoarticular elbow muscles (the lateral head of the triceps and brachioradialis). The EMG was recorded using the Delsys Bagnoli 8 electromyography system (Boston, MA). The skin was cleaned with alcohol and prepared with an abrasive gel to increase conductance. After the abrasive gel was removed with cotton wool, conductive gel was applied to the electrodes, which were secured to the skin with tape. Electrode placement was chosen to maximize signal while minimizing crosstalk from adjacent muscles. EMG signals were analog band-pass filtered between 20 and 450 Hz (in the Delsys Bagnoli EMG system) and then sampled at 2 kHz.

### Paradigm

With the exception of the pulse phase, in which a series of force pulses was applied to the handle of the robot while the hand was stationary, participants made 12 cm center-out reaching movements to one of four targets located at 0°, 90°, 180° and 270°. Visual feedback consisted of the home position (0.7 cm radius disk), the four targets (0.7 cm radius rings), a set of red lines connecting each target to the home position and the hand cursor (0.5 cm radius disk) (Fig. 1b). The four targets, the home position and the connecting lines were displayed at all times, except during the pulse phase. Each trial started when the hand cursor had remained within 0.3 cm of the home position at a speed below 0.5 cm/s for 100 ms. One of the targets was then filled in, followed 500 ms later by an auditory tone, which cued the participant to initiate their movement. The trial ended when the hand cursor had remained within 0.3 cm of the target position for 500 ms. If the peak speed of the movement was less than 40 cm/s or greater than 60 cm/s, a low-frequency auditory tone was played, and a ‘Too Slow’ or ‘Too Fast’ message was displayed, respectively. At the end of each trial, the hand was passively returned to the home position by the robotic manipulandum.

Participants were assigned to either a stiff group (*n* = 12), a relaxed group (*n* = 12) or a control group (*n* = 12). Each experiment began with a training phase to familiarize participants with the passive dynamics of the robot and the requirements of the task. Participants then performed a pre-exposure phase consisting of 100 null-field trials (20 blocks), a pulse phase consisting of 50 trials (stiff and relaxed groups only), a post-pulse phase consisting of four null-field trials, an exposure phase consisting of 410 force-field trials (83 blocks) and a post-exposure phase consisting of 100 null-field trials (20 blocks). Each block of five trials consisted of one null or field trial to each target and one channel trial to the 90° target. We only applied channel trials for one of the four targets (the 90° target for all participants to allow comparison) to provide enough force-field trials for learning without making the experiment excessively long. The order of trials within a block was pseudorandom, except that a channel trial never occurred on the first trial of a block. The force field direction was fixed for each participant but counterbalanced across participants.

The pulse phase was designed to increase muscle co-contraction in the stiff group and decrease muscle co-contraction in the relaxed group. Each trial started with the hand in the home position. A single force pulse (5 N for 500 ms) was then applied to the hand. The 50 force pulses (one for each trial) ranged uniformly in direction over 360° and were presented in a pseudorandom order. Participants in the stiff group were instructed to keep the handle positioned within a border (1.5 cm radius ring) around the home position by co-contracting the muscles of their limb (Fig. 1b). Participants in the relaxed group were instructed to let their hand move freely by relaxing the muscles of their limb. After the force pulse had ended, the handle of the manipulandum was passively returned to the home position in preparation for the next trial.

During the exposure phase, participants in the stiff group were instructed to maintain their arm stiff and to not deviate from the red lines connecting the home position to the target (described above), and participants in the relaxed group were instructed to relax their arm. The control group did not perform the pulse phase and were not given any explicit instructions regarding muscle co-contraction. The exact instructions given to participants can be found in the Supplementary Information.

Participants were given a 45 second rest break after the pre-exposure phase and after every 13 blocks (65 trials) in the exposure phase, with the exception of the end of the exposure phase, which was followed directly by the post-exposure phase. At the end of each rest break, participants in the stiff group were reminded that co-contracting their muscles may help them to make straight reaching movements, and participants in the relaxed group were reminded that if they relaxed their arm their movements would naturally become straighter. To mitigate the effects of time-dependent decay of memory, a channel trial did not occur in the first four movements after each rest break.

### Analysis

Data analysis was performed using MATLAB R2017b. Two measures of performance were computed. On exposure and null-field trials the maximum distance between the path of the hand and a straight line connecting the initial hand position and the target (maximum perpendicular error) was computed. On channel trials the percentage of the force field that was compensated for (adaptation) was computed by regressing the actual forces *f*_a_(*t*) generated by participants in the channel on the ideal forces *f*_i_(*t*) that would fully compensate for the forces on a force-field trial (defined in Eq. 1):

2 $${f}_{\text{a}}(t)=k\times {f}_{\text{i}}(t),$$

3 $${\rm{adaptation}}=k\times 100 \% ,$$

where *k* is the regression coefficient and *t* is the discrete time step of the channel trial. The offset of the regression was constrained to zero. For this analysis, we used the portion of the movement where the hand speed was greater than 1 cm/s. At all stages of the experiment the ideal force term *f*_i_(*t*) refers to the force required to fully compensate for the force field present in the exposure phase. Therefore, adaptation in the pre-exposure phase prior to learning should be close to zero. To combine maximum perpendicular error (MPE) and adaptation data across participants, data from individual participants was sign adjusted according to the direction of the field they were trained on.

To quantify oscillations on individual channel trials, we calculated an oscillation index that was the path length of each force profile (from when the hand speed exceeded 1 cm/s to when it dropped below 1 cm/s), where a greater path length indicates a more oscillatory profile. To control for differences in movement duration and adaptation level, each force profile was first normalized in amplitude by dividing by the force range (max - min) and normalized in time between 0 and 1.

The raw EMG signal was bandpass filtered (30–500 Hz) using a 10^th^-order Butterworth filter implemented with MATLAB’s filtfilt function and then full-wave rectified. We examined the EMG over two periods: an early period comprised of feedforward signals and a later period comprised of both feedforward and feedback signals. These periods were defined separately for the pulse phase and for reaching movements. In the pulse phase, the early period was taken from 200 ms before the perturbation onset to the perturbation onset, and the later period was taken from the perturbation onset to 130 ms after the perturbation onset. During reaching movements, the early period was taken from 200 ms before movement onset to 130 ms after movement onset. This period can be used as a measure of the feedforward muscle activation as feedback components of EMG are first detectable around 130 ms after movement onset when the hand is smoothly perturbed by a velocity-dependent force field^16^. The later period was taken from 130 ms to 400 ms after movement onset. To normalize EMG traces across the muscles, we first computed for each muscle the mean EMG over the early period for the non-channel trials in the pre-exposure phase. We then normalized the EMG trace for each trial by dividing the trace by the corresponding mean for that muscle^19^. Note that this only scales the EMG traces for each muscle and does not affect the temporal profile. A global measure of muscle activity was then computed for each trial by calculating the difference between the summed EMG across muscles (first averaged across time points on a trial) and the summed pre-exposure EMG across muscles (first averaged across time points and trials for the pre-exposure phase). Accordingly, negative global EMG signifies reduced activity relative to the pre-exposure phase, whereas positive global EMG signifies increased activity relative to the pre-exposure phase. For plotting purposes, EMG waveforms were time normalized by resampling 1000 data points between movement onset and movement end on each trial for each participant. Movement onset was defined as the time the cursor first moved 0.3 cm from the center of the home position. Movement end was defined as the time the cursor was first within 0.3 cm of the center of the target position. Each resampled data point was assigned a time point linearly spaced between 0 and the mean movement duration across participants.

To identify differences in global EMG, MPE and adaptation between the groups, between-subjects ANOVAs, mixed-design ANOVAs and unpaired *t*-tests were performed. We also performed linear regression between global EMG, adaptation and oscillation index. All statistical tests were two-sided with significance set to P < 0.05. Data are reported as mean ± standard error of the mean (s.e.m.).

## Results

**Figure 2:**
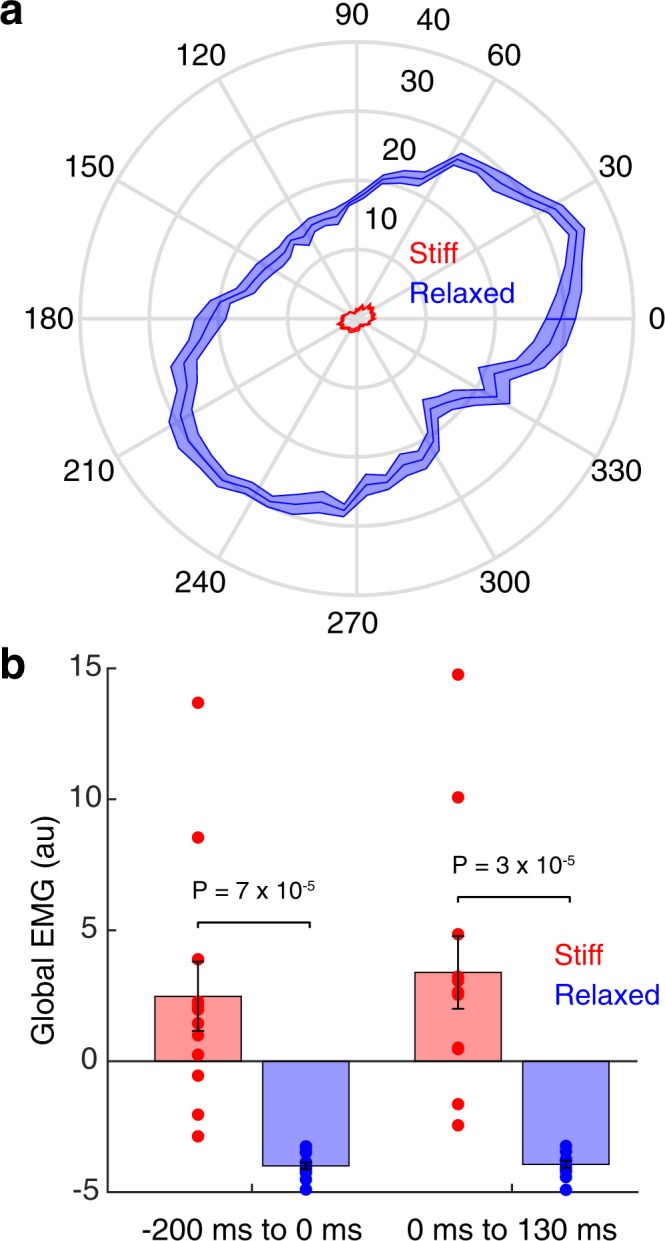
Performance in the force pulse phase. (**a**) Polar plot of the maximum distance (cm) the hand moved as a function of the force pulse direction. Data show mean ± s.e.m. across participants. (**b**) Global EMG before (left) and after (right) the force pulse for the stiff (red) and relaxed (blue) groups. Data show mean ± s.e.m. across participants.

Participants started the experiment by making center-out reaching movements in a null field (pre-exposure phase). Then, in the stiff and relaxed groups, voluntary changes to muscle co-contraction were elicited by instructing participants to resist or comply with a sequence of force pulses while the hand was stationary (pulse phase). Figure 2a shows the maximum distance moved by the hand following force pulses in different directions. The maximum distance moved by the hand was smaller in the stiff group compared with the relaxed group (stiff: 1.7 cm ± 0.1, relaxed: 23.2 ± 0.9, two-tailed unpaired *t*-test, *t*_22_ = 22.81, P = 8 × 10^−17^). This was due to greater muscle co-contraction, as confirmed by global EMG (stiff: 2.9 ± 1.4, relaxed: −4.0 ± 0.1, mixed-design ANOVA, F_1,22_ = 25.74, P = 4 × 10^−5^), both in the period preceding the force pulse (Fig. 2b; stiff: 2.5 ± 1.3, relaxed: −4.0 ± 0.1, two-tailed unpaired *t*-test, *t*_22_ = 4.87, P = 7 × 10^−5^) and in the period following pulse onset (Fig. 2b; stiff: 3.4 ± 1.4, relaxed: −3.9 ± 0.1, two-tailed unpaired *t*-test, *t*_22_ = 5.26, P = 3 × 10^−5^). The variability of global EMG was greater for the stiff group (Fig. 2b). This is most likely because the relaxed group are bounded by an EMG of zero, whereas the stiff group can stiffen up to different extents due to different bounds across participants. Note that because joint impedance increases faster than force variability as a function of muscle co-contraction^24^, co-contraction has a net stabilizing effect on endpoint kinematics^25,43^. The pulse phase was omitted for the control group.

### Electromyography

**Figure 3:**
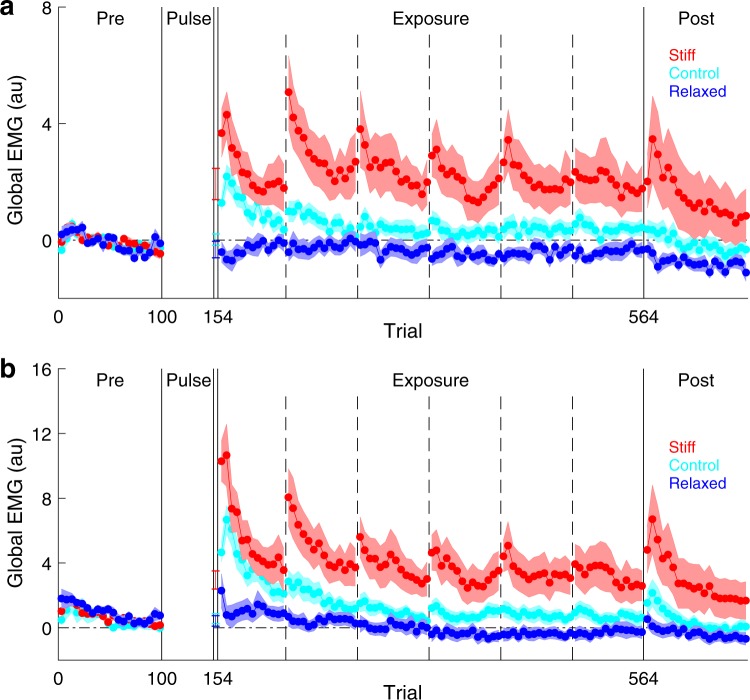
Global EMG over the course of the experiment. (**a**,**b**) Global EMG, which is a proxy for stiffness, was calculated on each trial and plotted as an average of a block of five trials. Data show mean ± s.e.m. across participants in the stiff (red), relaxed (blue) and control (cyan) groups. Vertical dashed lines indicate rest breaks. (**a**) Global EMG from −200 ms to 130 ms relative to movement onset. (**b**) Global EMG from 130 ms to 400 ms relative to movement onset.

After the pulse phase (stiff and relaxed groups) or the pre-exposure phase (control group), participants performed reaching movements in a velocity-dependent curl field (exposure phase). Figure 3 shows global EMG for all groups over the course of the experiment, split into periods separated by rest breaks (single-participant data shown in Supplementary Figs 1 and 2). To identify differences in muscle co-contraction between the groups during the exposure phase, we performed a mixed-design ANOVA with stage of exposure (first vs. second half) and period of movement (early vs. later period) as within-subject factors and global EMG as the response variable. We found a main effect of group (mixed-design ANOVA, F_2,33_ = 8.59, P = 0.001) but no interaction between group and stage of exposure (mixed-design ANOVA, F_2,33_ = 1.58, P = 0.220). We therefore combined data from the first and second halves of the exposure phase. Importantly, global EMG was greater in the stiff group than the relaxed group, both in the early period of the movement (Fig. 3a; stiff: 2.3 ± 0.8, relaxed: −0.4 ± 0.2, two-tailed unpaired *t*-test, *t*_22_ = 3.42, P = 0.002) and in the later period of the movement (Fig. 3b; stiff: 4.1 ± 1.1, relaxed: 0.1 ± 0.3, two-tailed unpaired *t*-test, *t*_22_ = 3.44, P = 0.002). Global EMG was also greater in the stiff group than the control group, both in the early period of the movement (Fig. 3a; control: 0.5 ± 0.2, two-tailed unpaired *t*-test, *t*_22_ = 2.28, P = 0.033) and in the later period of the movement (Fig. 3b; control: 1.5 ± 0.4, two-tailed unpaired *t*-test, *t*_22_ = 2.20, P = 0.038). Finally, Global EMG was greater in the control group than the relaxed group, both in the early period of the movement (Fig. 3a; two-tailed unpaired *t*-test, *t*_22_ = 3.15, P = 0.005) and in the later period of the movement (Fig. 3b; two-tailed unpaired *t*-test, *t*_22_ = 2.70, P = 0.013). Therefore, as expected, the stiff group exhibited the greatest degree of muscle co-contraction, followed by the control group and then the relaxed group.

**Figure 4:**
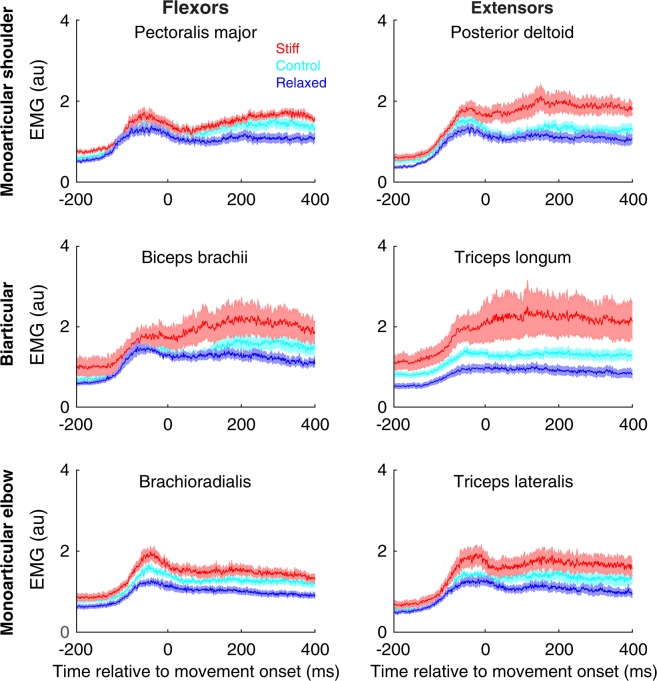
Muscle activity during the first half of the exposure phase. The EMG is averaged across all directions on force field trials. The period after movement onset has been time normalized. The time from 0 ms to 400 ms corresponds to approximately 64% of the mean movement duration across participants. The period before movement onset is shown in real time. Data show mean ± s.e.m. across all participants in the stiff (red), relaxed (blue) and control (cyan) groups.

Figure 4 shows the average EMG of the stiff (red), relaxed (blue) and control (cyan) groups during the first half of the exposure phase. This shows an increase in EMG both prior to and during movement in many of the muscles. Overall, a clear voluntary increase in EMG can be seen for the stiff group compared with the relaxed group. This increase is seen in all muscles pairs, suggesting that muscle co-contraction is not specific to a single joint. The EMG of the control group is intermediate between the stiff and relaxed groups.

### Movement Analysis

**Figure 5:**
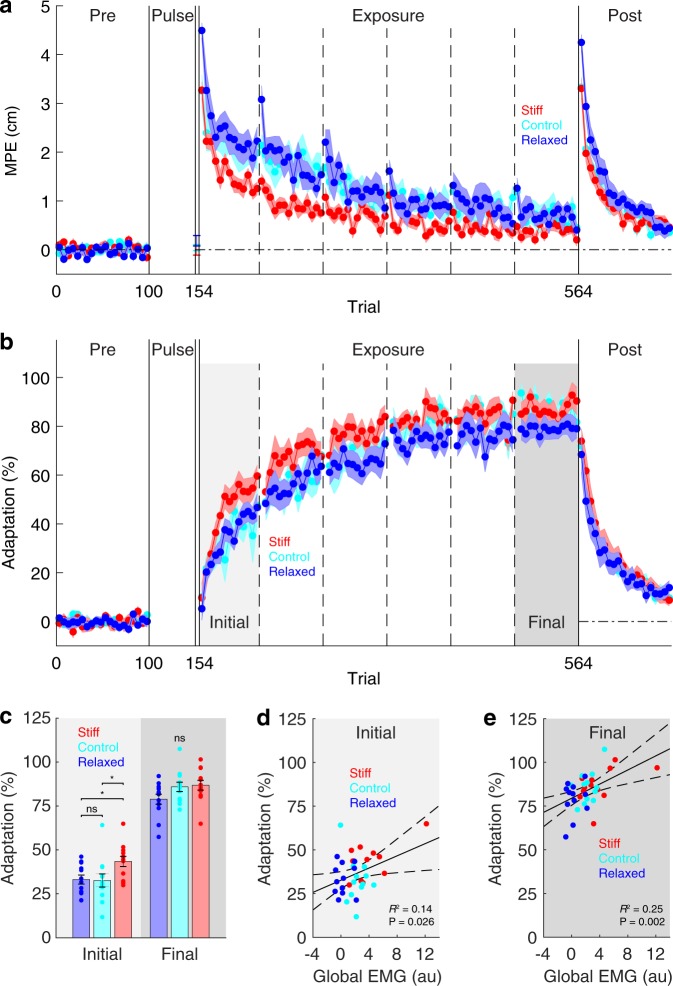
Kinematic and dynamic adaptation over the course of the experiment. (**a**) Maximum perpendicular error (MPE) was calculated on each trial and plotted as an average of a block of five trials. Data show mean ± s.e.m. across participants in the stiff (red), relaxed (blue) and control (cyan) groups. Vertical dashed lines indicate rest breaks. (**b**) Is the same as (**a**) but for adaptation measured on channel trials. (**c**) Mean ± s.e.m. initial adaptation (first 13 blocks of the exposure phase) and final adaptation (last 13 blocks of the exposure phase) for all groups. Asterisks indicate significant (*P < 0.05), ns indicates not significant. (**d**,**e**) Least-squares regression between each participant’s global EMG (first 13 blocks of the exposure phase, early and later periods of movement combined) and their initial adaptation (**d**) and their final adaptation (**e**). Dashed curves show 95% confidence intervals for the fitted linear model. Data from all groups have been pooled.

In the pre-exposure phase, reaching movements were relatively straight in all groups, as demonstrated by small MPEs (Fig. 5a; single-participant data shown in Supplementary Fig. 3). During the exposure phase, there was no difference in peak velocity between the groups (stiff: 48.8 ± 0.4 cm/s, relaxed: 48.4 ± 0.5, control: 48.0 ± 0.5, between-subjects ANOVA, F_2,33_ = 0.61, P = 0.549). Therefore, each group experienced similar forces during learning. In the first block (5 trials) of the exposure phase, all groups showed large deviations from a straight line, and these deviations were different between the groups (between-subjects ANOVA, F_2,33_ = 9.94, P = 4 × 10^−4^). Importantly, MPE was, as expected, smaller in the stiff group compared with the relaxed group (stiff: 3.3 cm ± 0.2, relaxed: 4.5 ± 0.2, two-tailed unpaired *t*-test, *t*_22_ = 5.42, P = 2 × 10^−5^). Furthermore, MPE was also smaller in the control group compared with the relaxed group (control: 3.2 ± 0.3, two-tailed unpaired *t*-test, *t*_22_ = 3.49, P = 0.002). However, there was no difference in MPE between the stiff group and the control group (two-tailed unpaired *t*-test, *t*_22_ = 0.03, P = 0.977). As the exposure phase progressed, MPE decreased in all groups. By the final 13 blocks (65 trials) of the exposure phase, there was no difference in MPE between the groups (stiff: 0.4 cm ± 0.1, relaxed: 0.7 ± 0.1, control: 0.7 ± 0.2, between-subjects ANOVA, F_2,33_ = 1.94, P = 0.160). In the null field of the post-exposure phase, MPE rose again, demonstrating after-effects.

In the relaxed group, increases in MPE can be seen at the start of each rest break (Fig. 5a; vertical dashed lines) when participants were reminded that their movements would naturally become straighter if they relaxed their arm. In the stiff group, changes in MPE cannot be seen at the start of each rest break. This is likely because decreases in MPE due to increased co-contraction are offset by increases in MPE due to forgetting of learning during rest breaks.

### Force compensation

In the pre-exposure phase, adaptation measured on channel trials was, as expected, close to zero in all groups (Fig. 5b; single-participant data shown in Supplementary Fig. 4). During exposure, adaptation increased in all groups, eventually reaching asymptote. To assess learning, we compared initial (first 13 blocks of the exposure phase) and final (last 13 blocks of the exposure phase) adaptation between the groups. This revealed a main effect of group (mixed-design ANOVA, F_2,33_ = 3.75, P = 0.034) and no interaction (mixed-design ANOVA, F_2,33_ = 2.35, P = 0.111). Initial adaptation was greater in the stiff group than both the relaxed group (Fig. 5c; stiff: 43.5 ± 2.9, relaxed: 33.2 ± 2.5, two-tailed unpaired *t*-test, *t*_22_ = 2.68, P = 0.014) and the control group (Fig. 5c; control: 32.6 ± 3.7, two-tailed unpaired *t*-test, *t*_22_ = 2.31, P = 0.031). However, there was no difference in initial adaptation between the relaxed group and the control group (Fig. 5c; two-tailed unpaired *t*-test, *t*_22_ = 0.12, P = 0.904). Final adaptation was not significantly different between the groups (Fig. 5c; stiff: 86.9 ± 2.8, relaxed: 78.9 ± 2.9, control: 85.9 ± 2.7, between-subjects ANOVA, F_2,33_ = 2.49, P = 0.099).

We examined data at the individual level pooling across all three groups. This revealed a significant linear relationship between a participant’s global EMG (first 13 blocks of the exposure phase, early and later periods of the movement combined) and both their initial adaptation (Fig. 5d; *R*^2^ = 0.14, β = 1.74, *F*-test, F_1,34_ = 5.45, P = 0.026) and their final adaptation (Fig. 5e; *R*^2^ = 0.25, β = 2.02, *F*-test, F_1,34_ = 11.17, P = 0.002). To rule out the possibility that muscle co-contraction influences adaptation by increasing/decreasing oscillations on channel trials, we performed multiple linear regression with global EMG and oscillation index (see methods) as predictors and adaptation as the response variable. Importantly, oscillation index was not a significant predictor of either initial adaptation (β = −0.17, two-tailed *t*-test, *t*_33_ = 0.04, P = 0.971) or final adaptation (β = −7.58, two-tailed *t*-test, *t*_33_ = 1.00, P = 0.327). In contrast, global EMG was a significant predictor of both initial adaptation (β = 1.75, two-tailed *t*-test, *t*_33_ = 2.19, P = 0.036) and final adaptation (β = 2.25, two-tailed *t*-test, *t*_33_ = 3.24, P = 0.003). Therefore, the relationship between global EMG and adaptation cannot be explained in terms of increased/decreased oscillations on channel trials due to muscle co-contraction.

## Discussion

We examined force-field adaptation under increased, decreased and baseline levels of muscle co-contraction. Participants were randomly assigned to either a stiff, relaxed or control group. The stiff and relaxed groups were pretrained using a sequence of force pulses to either increase (stiff group) or decrease (relaxed group) muscle co-contraction in the presence of perturbations. The control group was not pretrained. All groups performed reaching movements in a velocity-dependent curl field. We assessed learning by measuring adaptation, that is predictive force compensation using channels trials. In the initial stage of exposure, the stiff group adapted more than the relaxed and control groups, despite experiencing smaller kinematic errors, an important training signal for error-based learning. Furthermore, at the individual level, muscle co-contraction was significantly correlated with adaptation, both in the initial and final stage of exposure. These results show that, in addition to improving kinematic accuracy, muscle co-contraction also increases the rate of acquisition of an internal model.

There are a number of reasons why increased muscle co-contraction could lead to an increase in the rate of dynamic motor learning. First, when motor errors are experienced, the internal model is updated with respect to the actual states visited rather than the intended states, a process termed motion-referenced learning^21^. Consequently, any intervention that increases the overlap between the actual motions experienced and the motion required to reach the target, such as increased muscle co-contraction, could increase the rate of adaptation. Second, error sensitivity is greater for smaller errors^34,36,44^. This could explain why muscle co-contraction accelerates adaptation, despite decreasing the size of errors. In a causal inference framework^45^, larger errors are more likely to have been caused by factors extrinsic to the motor plant and so should be discounted to a greater extent. Consequently, larger errors do not guarantee greater learning. Indeed, single-trial adaptation (the product of error sensitivity and error magnitude) appears to be non-monotonic with a peak at an intermediate error magnitude^46,47^. This non-monotonic relationship can be appreciated in studies of error augmentation. For example, during reach adaptation to a visuomotor rotation, the rate of learning almost doubles when visual errors are amplified by a gain of 2^48^. However, when visual errors are amplified by a gain of 3, the rate of learning returns to the level associated with veridical visual feedback^48^. Given this non-monotonic relationship between adaptation and error magnitude, it is possible that muscle co-contraction increases the rate of adaptation by reducing errors to a size that induces greater learning.

Previous studies of error sensitivity^16,34,36,44,46,47^ did not examine its dependence on muscle co-contraction. Therefore, it is possible that error sensitivity is a function of muscle co-contraction, such that as muscle co-contraction increases, single-trial adaptation is maximized by progressively smaller errors. For the sensorimotor system to implement such an error-sensitivity function, it would need to have knowledge of the effects of muscle co-contraction on the statistics of kinematic errors. Such knowledge has been postulated in normative models of impedance control, in which muscle co-contraction emerges as a strategy to minimize the expected cost of movement by reducing uncertainty in the forward dynamics model^8^. If error sensitivity were a function of muscle co-contraction, when kinematic errors are experienced, the sensorimotor system could appropriately assign credit between the forward dynamics model and sensorimotor noise, controlling for the level of muscle co-contraction. Interestingly, the forward dynamics model, which maps current states and motor commands to next states, is also a function of muscle co-contraction (a state determined by a motor command), and so it is an open question to what degree adaptation under increased co-contraction generalizes to other co-contraction levels.

All participants were able to form an internal model of the force field, as demonstrated by adaptation on channel trials and after-effects in the post-exposure phase. This is consistent with previous studies that have demonstrated that people can adapt to viscous force fields that are superimposed on background loads^49,50^. It has even been suggested that people partition force fields into dynamic and static components^49^, which could allow velocity-dependent dynamics to be learned across a range of muscle load states. However, not all muscle states appear to be available to the internal model. For example, after recovery from fatigue, muscles initially generate excessive force, as if the internal model is still producing motor commands appropriate for fatigued muscles^51^.

In our study, although participants made movements in four directions, we only measured adaptation for one direction to limit the length of the experiment. Given that endpoint stiffness is in general anisotropic^10,52,53^, adaptation may similarly have been anisotropic, giving rise to an adaptation ellipse. Moreover, the geometry (size, shape, orientation) of this adaptation ellipse, like the geometry of the stiffness ellipse^10,52,53^, may be modifiable, allowing the rate of adaptation to be modulated in a direction-dependent manner. For example, by orienting the major axis of the stiffness ellipse in the direction of greatest unpredictability in the environment, it may be possible to maximize the rate of adaptation in the direction it is needed most: where errors are largest.

It remains an open question whether muscle co-contraction can modulate adaptation independently of the errors experienced. Feedback error learning (FEL)^31,54^ provides one possible mechanism by which this could occur. According to FEL, the feedback response to error acts as a teaching signal for motor adaptation. Consistent with FEL, single-trial adaptation has been shown to correlate with the magnitude of the feedback response^14^. Given that feedback gains scale with muscle activity^55^ and feedback responses are potentiated when muscles are co-contracted^56–58^, adaptation to an error of a given magnitude should be larger when muscles are co-contracted.

In conclusion, we have shown that muscle co-contraction accelerates dynamic motor learning, despite reducing kinematic errors, which are the main driving force behind adaptation. Muscle co-contraction therefore improves both kinematic accuracy and dynamic learning, simultaneously enhancing present and future motor performance. The modifiable nature of muscle co-contraction suggests that the rate of motor adaptation can be actively modulated.

## Electronic supplementary material

Supplementary Information

## Data Availability

Data that support the findings of this study are available from the corresponding author on request.

## References

[1] Franklin DW, Osu R, Burdet E, Kawato M, Milner TE (2003). Adaptation to stable and unstable dynamics achieved by combined impedance control and inverse dynamics model. J. Neurophysiol..

[2] Osu R, Burdet E, Franklin DW, Milner TE, Kawato M (2003). Different mechanisms involved in adaptation to stable and unstable dynamics. J. Neurophysiol..

[3] Shadmehr R, Mussa-Ivaldi FA (1994). Adaptive representation of dynamics during learning of a motor task. J. Neurosci..

[4] Lackner JR, Dizio P (1994). Rapid adaptation to Coriolis force perturbations of arm trajectory. J. Neurophysiol..

[5] Conditt MA, Gandolfo F, Mussa-Ivaldi FA (1997). The motor system does not learn the dynamics of the arm by rote memorization of past experience. J. Neurophysiol..

[6] Donchin O, Francis JT, Shadmehr R (2003). Quantifying generalization from trial-by-trial behavior of adaptive systems that learn with basis functions: theory and experiments in human motor control. J. Neurosci..

[7] Burdet E (2006). Stability and motor adaptation in human arm movements. Biol. Cybern..

[8] Mitrovic D, Klanke S, Osu R, Kawato M, Vijayakumar S (2010). A computational model of limb impedance control based on principles of internal model uncertainty. PLoS One.

[9] Hogan N (1985). The mechanics of multi-joint posture and movement control. Biol. Cybern..

[10] Burdet E, Osu R, Franklin DW, Milner TE, Kawato M (2001). The central nervous system stabilizes unstable dynamics by learning optimal impedance. Nature.

[11] Lametti DR, Houle G, Ostry DJ (2007). Control of movement variability and the regulation of limb impedance. J. Neurophysiol..

[12] Osu R (2004). Optimal impedance control for task achievement in the presence of signal-dependent noise. J. Neurophysiol..

[13] Milner TE, Franklin DW (2005). Impedance control and internal model use during the initial stage of adaptation to novel dynamics in humans. J. Physiol..

[14] Albert ST, Shadmehr R (2016). The Neural Feedback Response to Error As a Teaching Signal for the Motor Learning System. J. Neurosci..

[15] Sing GC, Joiner WM, Nanayakkara T, Brayanov JB, Smith MA (2009). Primitives for motor adaptation reflect correlated neural tuning to position and velocity. Neuron.

[16] Franklin DW (2008). CNS Learns Stable, Accurate, and Efficient Movements Using a Simple Algorithm. Journal of Neuroscience.

[17] Darainy M, Ostry DJ (2008). Muscle cocontraction following dynamics learning. Exp. Brain Res..

[18] Thoroughman KA, Shadmehr R (1999). Electromyographic correlates of learning an internal model of reaching movements. J. Neurosci..

[19] Franklin S, Wolpert DM, Franklin DW (2012). Visuomotor feedback gains upregulate during the learning of novel dynamics. J. Neurophysiol..

[20] Huang HJ, Kram R, Ahmed AA (2012). Reduction of metabolic cost during motor learning of arm reaching dynamics. J. Neurosci..

[21] Gonzalez Castro LN, Monsen CB, Smith MA (2011). The binding of learning to action in motor adaptation. PLoS Comput. Biol..

[22] Sanger TD (1994). Neural network learning control of robot manipulators using gradually increasing task difficulty. IEEE Trans. Rob. Autom..

[23] Kagerer FA, Contreras-Vidal JL, Stelmach GE (1997). Adaptation to gradual as compared with sudden visuo-motor distortions. Exp. Brain Res..

[24] Selen LPJ, Beek PJ, van Dieën JH (2005). Can co-activation reduce kinematic variability? A simulation study. Biol. Cybern..

[25] Gribble PL, Mullin LI, Cothros N, Mattar A (2003). Role of cocontraction in arm movement accuracy. J. Neurophysiol..

[26] Wong J, Wilson ET, Malfait N, Gribble PL (2009). Limb stiffness is modulated with spatial accuracy requirements during movement in the absence of destabilizing forces. J. Neurophysiol..

[27] Wu HG, Miyamoto YR, Gonzalez Castro LN, Ölveczky BP, Smith MA (2014). Temporal structure of motor variability is dynamically regulated and predicts motor learning ability. Nat. Neurosci..

[28] Kao MH, Doupe AJ, Brainard MS (2005). Contributions of an avian basal ganglia–forebrain circuit to real-time modulation of song. Nature.

[29] Tumer EC, Brainard MS (2007). Performance variability enables adaptive plasticity of ‘crystallized’ adult birdsong. Nature.

[30] Charlesworth JD, Warren TL, Brainard MS (2012). Covert skill learning in a cortical-basal ganglia circuit. Nature.

[31] Kawato M, Furukawa K, Suzuki R (1987). A hierarchical neural-network model for control and learning of voluntary movement. Biol. Cybern..

[32] Thoroughman KA, Shadmehr R (2000). Learning of action through adaptive combination of motor primitives. Nature.

[33] Milner TE, Hinder MR (2006). Position information but not force information is used in adapting to changes in environmental dynamics. J. Neurophysiol..

[34] Fine MS, Thoroughman KA (2007). Trial-by-trial transformation of error into sensorimotor adaptation changes with environmental dynamics. J. Neurophysiol..

[35] Körding KP, Wolpert DM (2004). Bayesian integration in sensorimotor learning. Nature.

[36] Wei K, Körding K (2009). Relevance of error: what drives motor adaptation?. J. Neurophysiol..

[37] Blakemore SJ, Wolpert DM, Frith CD (1998). Central cancellation of self-produced tickle sensation. Nat. Neurosci..

[38] Blakemore S-J, Wolpert D, Frith C (2000). Why can’t you tickle yourself?. Neuroreport.

[39] Shergill SS, Bays PM, Frith CD, Wolpert DM (2003). Two eyes for an eye: the neuroscience of force escalation. Science.

[40] Oldfield RC (1971). The assessment and analysis of handedness: the Edinburgh inventory. Neuropsychologia.

[41] Howard IS, Ingram JN, Wolpert DM (2009). A modular planar robotic manipulandum with end-point torque control. J. Neurosci. Methods.

[42] Scheidt RA, Reinkensmeyer DJ, Conditt MA, Rymer WZ, Mussa-Ivaldi FA (2000). Persistence of motor adaptation during constrained, multi-joint, arm movements. J. Neurophysiol..

[43] Selen LPJ, Franklin DW, Wolpert DM (2009). Impedance control reduces instability that arises from motor noise. J. Neurosci..

[44] Marko MK, Haith AM, Harran MD, Shadmehr R (2012). Sensitivity to prediction error in reach adaptation. J. Neurophysiol..

[45] Körding KP (2007). Causal inference in multisensory perception. PLoS One.

[46] Robinson FR, Noto CT, Bevans SE (2003). Effect of visual error size on saccade adaptation in monkey. J. Neurophysiol..

[47] Herzfeld DJ, Vaswani PA, Marko MK, Shadmehr R (2014). A memory of errors in sensorimotor learning. Science.

[48] Patton JL, Wei YJ, Bajaj P, Scheidt RA (2013). Visuomotor learning enhanced by augmenting instantaneous trajectory error feedback during reaching. PLoS One.

[49] Kurtzer I, DiZio PA, Lackner JR (2005). Adaptation to a novel multi-force environment. Exp. Brain Res..

[50] Liu J, Reinkensmeyer DJ (2007). Motor adaptation to a small force field superimposed on a large background force. Exp. Brain Res..

[51] Takahashi CD (2006). Effect of muscle fatigue on internal model formation and retention during reaching with the arm. J. Appl. Physiol..

[52] Franklin DW, So U, Kawato M, Milner TE (2004). Impedance control balances stability with metabolically costly muscle activation. J. Neurophysiol..

[53] Franklin DW (2007). Endpoint stiffness of the arm is directionally tuned to instability in the environment. J. Neurosci..

[54] Kawato, M. Feedback-Error-Learning Neural Network for Supervised Motor Learning. In *Advanced Neural Computers* (ed. Eckmiller, R.) 365–372 (North-Holland, 1990).

[55] Pruszynski JA, Kurtzer I, Lillicrap TP, Scott SH (2009). Temporal evolution of ‘automatic gain-scaling’. J. Neurophysiol..

[56] Doemges F, Rack PM (1992). Task-dependent changes in the response of human wrist joints to mechanical disturbance. J. Physiol..

[57] Akazawa K, Milner TE, Stein RB (1983). Modulation of reflex EMG and stiffness in response to stretch of human finger muscle. J. Neurophysiol..

[58] Krutky MA, Ravichandran VJ, Trumbower RD, Perreault EJ (2010). Interactions between limb and environmental mechanics influence stretch reflex sensitivity in the human arm. J. Neurophysiol..

