# Supplementary material for: Increasing muscle co-contraction speeds up internal model acquisition during dynamic motor learning
Source: Sci Rep. 2018 Nov 5;8:16355. doi: 10.1038/s41598-018-34737-5 (PMC6218508; doi:10.1038/s41598-018-34737-5)
Supplement: Supplementary file 1 — Supplementary Information [file 41598_2018_34737_MOESM1_ESM.pdf]

Increasing muscle co-contraction speeds up internal model acquisition during dynamic motor learning

Supplementary Information

James B. Heald*[1], David W. Franklin[2] & Daniel M. Wolpert[1]

[1]Computational and Biological Learning Lab, Department of Engineering, University of Cambridge, Cambridge, CB2 1PZ, United Kingdom
[2]Neuromuscular Diagnostics, Department of Sport and Health Sciences, Technical University of Munich, 80992 Munich, Germany

*Corresponding author: jbh40@cam.ac.uk

Instructions to participants

Instructions to the stiff group (provided prior to commencement of the pulse phase)

I'm now going to describe the next phase of the experiment. Each trial will start when you place the green cursor in the center of the white home position. A brief force will then be applied to the handle of the robot in a randomly chosen direction. This will only last for half a second. Your task will be to keep the handle as still as possible. To keep the handle still, you will need to stiffen up your arm by tensing the muscles in your hand, arm and chest. If on one trial you get pushed outside of the red ring that surrounds the white home position, you should try to stiffen up more on the next trial to help you stay within the ring. Ideally, you should keep the handle as still and as close to the center of the white home position as possible.

Once you have completed this phase of the experiment, all subsequent trials will be similar to the ones you've just done; that is, they will involve you making straight reaching movements to one of four targets. However, at some point your movements will be unexpectedly thrown off course by forces generated by the robot. Your task will be to keep your movements as straight as possible. In particular, you should try to stay as close as possible to the red lines that lead from the home position to the targets. You may find that stiffening up your arm will help you to do this. If at any point you feel as though your arm is too sore or tired, just let me know and you can have a rest break immediately. You will automatically get a rest break every 5 minutes though and ideally you should wait until then in order to allow the experiment to run smoothly.

Instructions to the relaxed group (provided prior to commencement of the pulse phase)

I'm now going to describe the next phase of the experiment. Each trial will start when you place the green cursor in the center of the white home position. A brief force will then be applied to the handle of the robot in a randomly chosen direction. This will only last for half a second. You should let your hand move freely to wherever the handle takes it. To do this, you should relax the muscles in your arm as much as possible so that you don't provide any resistance to the handle as it moves. Once the handle has stopped the robot will automatically bring you back to the central home position. Once again, you should keep your arm as relaxed as possible when this happens. Throughout all the trials of this phase you will see a red ring surrounding the white home position. This ring has no relevance and you should ignore it.

Once you have completed this phase of the experiment, all subsequent trials will be similar to the ones you've just done; that is, they will involve you making straight reaching movements to one of four targets. However, at some point your movements will be unexpectedly thrown off course by forces generated by the robot. Try not to stiffen up when this happens. If you relax your arm you will find that your movements will naturally become straighter as you adapt to the forces.

Instructions to the control group (provided prior to commencement of the experiment)

At some point during the experiment, your movements will be unexpectedly thrown off course by forces generated by the robot. When this happens, keep trying to reach the target, and you will find that you will naturally adapt to the forces over time.

Supplementary Figures

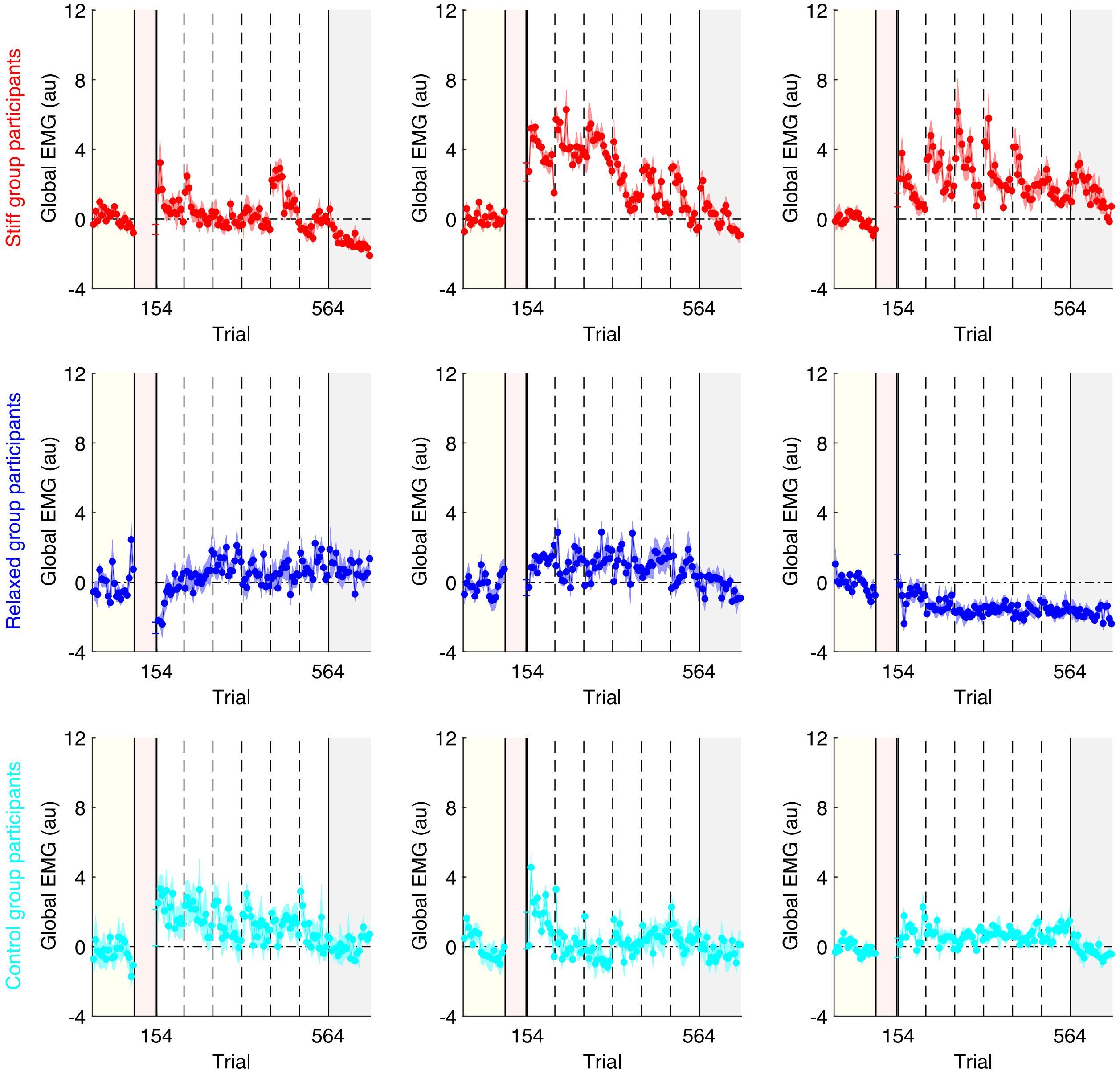

Supplementary Figure 1 | Global EMG in the early movement period over the course of the experiment. Global EMG was calculated on each trial from -200 ms to 130 ms relative to movement onset and plotted as mean ± s.e.m over a block of five trials. Each subplot shows data from an individual participant in either the stiff (red), relaxed (blue) or control (cyan) groups (the same nine participants are shown in Supplementary Fig. 1-4). Shaded regions indicate the pre-exposure (yellow), pulse (red), exposure (white), and post-exposure (gray) phases. Vertical dashed lines denote rest breaks.

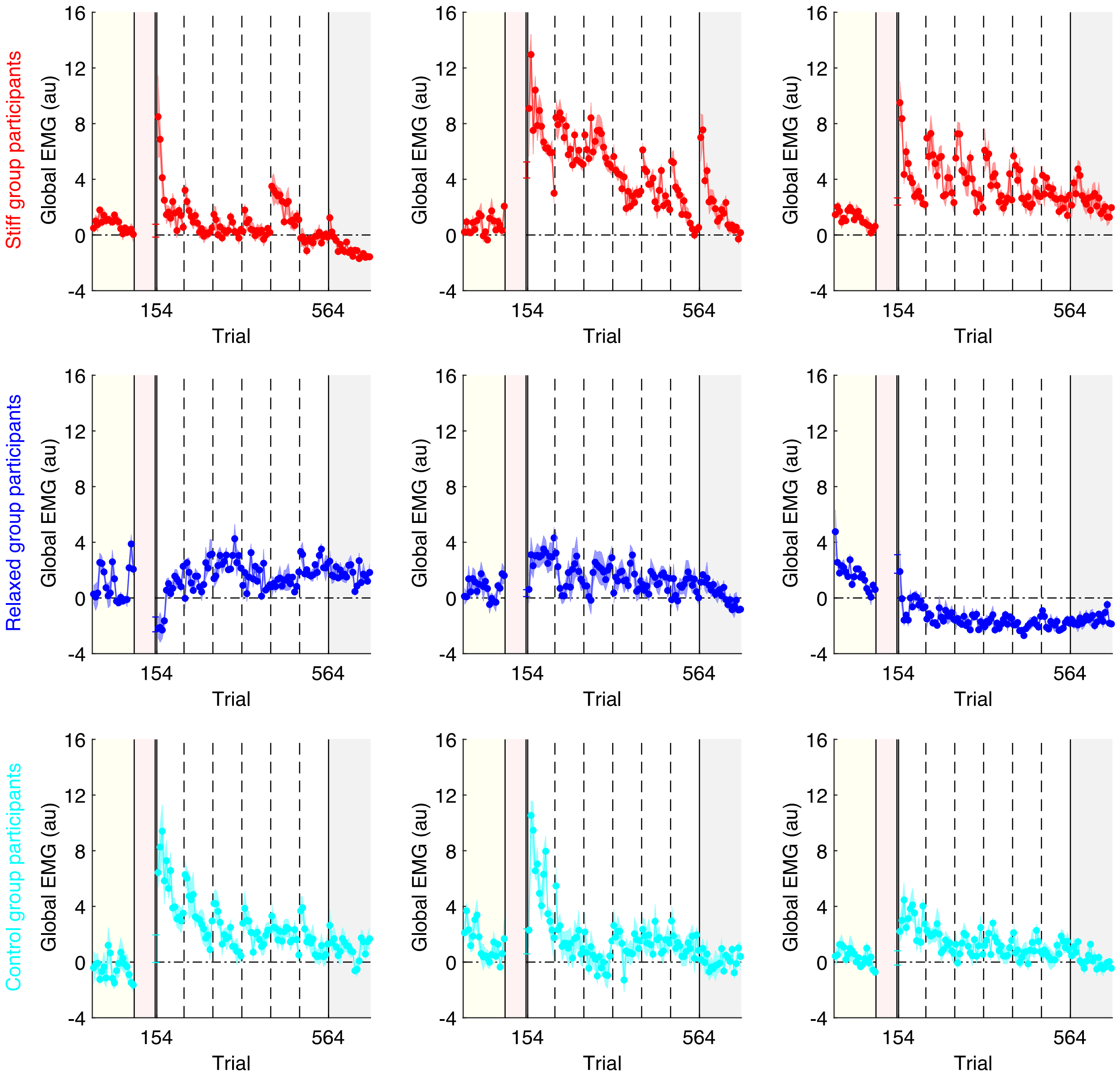

Supplementary Figure 2 | Global EMG in the later movement period over the course of the experiment. Global EMG was calculated on each trial from 130 ms to 400 ms relative to movement onset and plotted as mean ± s.e.m over a block of five trials. Each subplot shows data from an individual participant in either the stiff (red), relaxed (blue) or control (cyan) groups (the same nine participants are shown in Supplementary Fig. 1-4). Shaded regions indicate the pre-exposure (yellow), pulse (red), exposure (white), and post-exposure (gray) phases. Vertical dashed lines denote rest breaks.

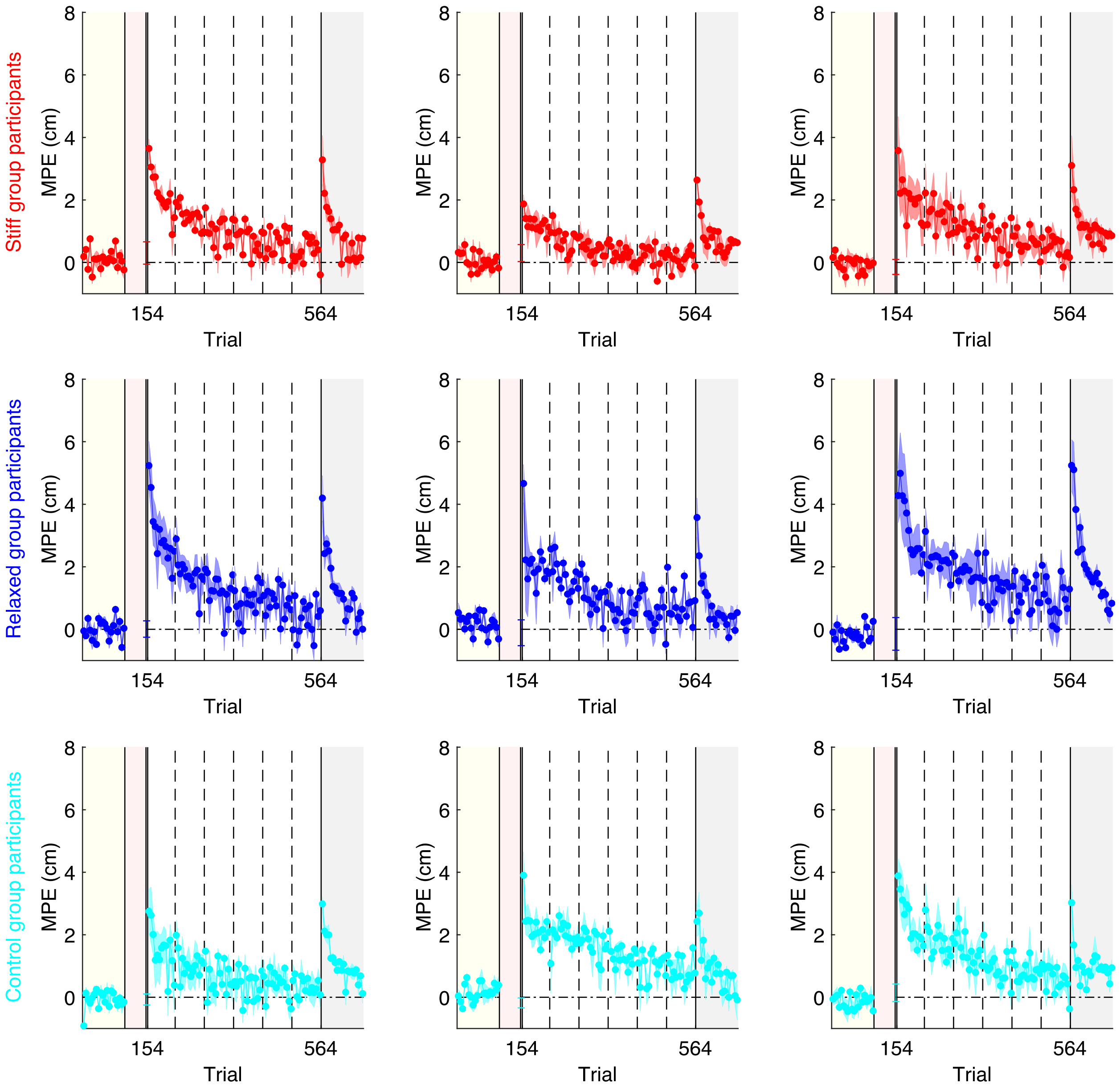

Supplementary Figure 3 | Kinematic adaptation over the course of the experiment. Maximum perpendicular error (MPE) was calculated on each trial and plotted as mean ± s.e.m over a block of five trials. Each subplot shows data from an individual participant in either the stiff (red), relaxed (blue) or control (cyan) groups (the same nine participants are shown in Supplementary Fig. 1-4). Shaded regions indicate the pre-exposure (yellow), pulse (red), exposure (white), and post-exposure (gray) phases. Vertical dashed lines denote rest breaks.

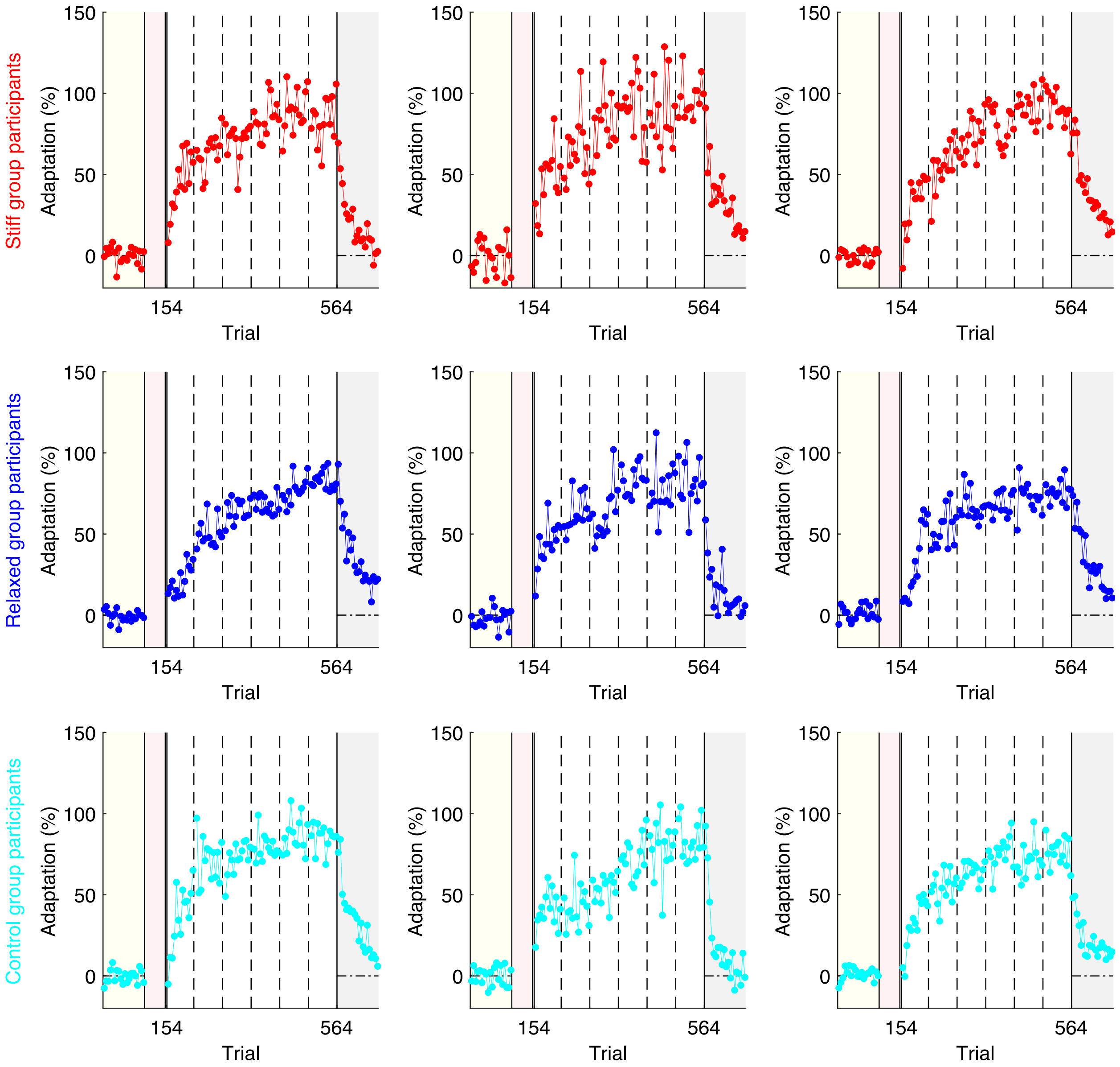

Supplementary Figure 4 | Dynamic adaptation over the course of the experiment. Adaptation measured on channel trials. Each subplot shows data from an individual participant in either the stiff (red), relaxed (blue) or control (cyan) groups (the same nine participants are shown in Supplementary Fig. 1-4). Shaded regions indicate the pre-exposure (yellow), pulse (red), exposure (white), and post-exposure (gray) phases. Vertical dashed lines denote rest breaks.
